# Allergic sensitisation and type‐2 inflammation is associated with new‐onset and persistent allergic disease

**DOI:** 10.1002/clt2.12240

**Published:** 2023-04-06

**Authors:** Viiu Blöndal, Fredrik Sundbom, Xingwu Zhou, Robert Movérare, Magnus P. Borres, Marieann Högman, Kjell Alving, Andrei Malinovschi, Christer Janson

**Affiliations:** ^1^ Department of Medical Sciences, Respiratory, Allergy and Sleep Research Uppsala University Uppsala Sweden; ^2^ Department of Medical Sciences Clinical Physiology Uppsala University Uppsala Sweden; ^3^ Thermo Fischer Scientific Uppsala Sweden; ^4^ Department of Women's and Children's Health Uppsala University Uppsala Sweden

**Keywords:** asthma, ENT, epidemiology

## Abstract

**Background:**

Allergic disease is common. The aim of this study was to look at the change in asthma and rhinitis over time and to characterise factors contributing to remission and persistence of disease.

**Methods:**

This cohort study included 255 individuals with or without asthma and or rhinitis that participated in a population survey and a follow‐up 10 years later. The participants were tested for allergic sensitisation, total IgE, multiplex allergen component analysis and type‐2 inflammatory markers: exhaled nitric oxide (F_E_NO), eosinophil cationic protein (ECP) and eosinophil‐derived neurotoxin (EDN).

**Results:**

Of the 132 healthy individuals, 112 remained healthy, 16 developed rhinitis, 4 asthma and rhinitis over the 10 years. Out of 82 subjects with rhinitis, 26 went into remission, 53 remained unchanged and 3 developed asthma in addition to rhinitis. None of the 41 participants with asthma and rhinitis went into remission. Subjects with persistent rhinitis and asthma had higher levels of total IgE (odds ratio [OR] 95% confidence interval [CI]: 6.16 [3.05–12.5]) at baseline and after 10 years, and F_E_NO and ECP at baseline (OR per log unit increase, 95% CI 5.21 [1.20–22.7] and 6.32 [1.52–26.4], respectively), compared with those that remained healthy. Subjects with persistent rhinitis were more likely to be sensitised to grass pollen and had higher total IgE levels than those that went into remission. Individuals with persistent asthma were more likely to be sensitised to tree pollen and furry animals than those with only persistent rhinitis (OR 95% CI: 3.50 [1.29–9.49] and 6.73 [2.00–22.6], respectively).

**Conclusion:**

IgE sensitisation and total IgE levels are associated with the persistence of rhinitis and asthma. Participants with persistent allergic disease had higher levels of allergen sensitisation and type 2 inflammation markers at baseline than those who remained healthy.

## 1 BACKGROUND

Allergic sensitisation and disease are common.^1^, ^2^, ^3^ Furthermore, many have a combination of rhinitis, asthma and eczema or all three.^4^, ^5^, ^6^ Not much information is available on how allergic disease and multimorbidity change over time and if there are predictors for possible remission and continued or new onset of disease.

Remission of asthma seems to be more likely in children than adults.^7^, ^8^ Factors previously negatively associated with remission of rhinitis and asthma seem to be heredity for allergy, allergen sensitisation and allergic multimorbidity.^9^ Persistent allergen sensitisation is associated with continued allergic disease^10^ and allergic sensitisation in childhood is a risk factor for developing allergic disease later in life.^9^, ^11^

It is unclear if type2 inflammation plays a role in persistent allergic diseases. Higher total immunoglobulin E (IgE) levels have previously been associated with elevated levels of positive specific IgE tests as well as allergic disease and multimorbidity.^12^, ^13^, ^14^ Elevated levels of exhaled nitric oxide (F_E_NO) are seen in allergic rhinitis and asthma but also in people with allergic multimorbidity.^15^ Higher levels of F_E_NO have been described as predictive of future rhinitis^16^ and asthma.^17^, ^18^ Eosinophil cationic protein (ECP) and eosinophil‐derived neurotoxin (EDN) are eosinophil activation markers, and higher levels of eosinophils have been described in both allergic sensitisation and allergic disease.^19^ Previous studies suggest that those with asthma or rhinitis have higher levels of eosinophilic inflammation, both in terms of ECP and EDN, compared with healthy individuals.^20^, ^21^ There is some evidence that ECP and EDN are associated with asthma severity.^22^ Information on eosinophil activation markers as predictors for developing asthma or rhinitis is scarce.

The aim of this study was to look at the change in allergic disease over time and to better characterise factors contributing to remission or persistent disease with the help of background factors, lung function, allergic sensitisation and type‐2 inflammation markers.

## 2 METHODS

### 2.1 Study population

**FIGURE 1:**
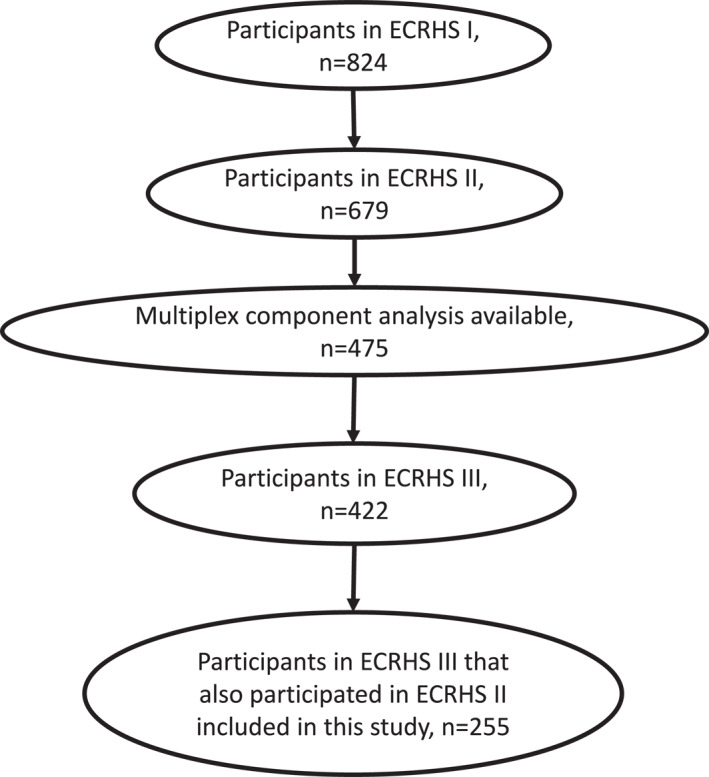
Study participants, European Community Respiratory Health Survey (ECRHS), part II and III.

**FIGURE 2:**
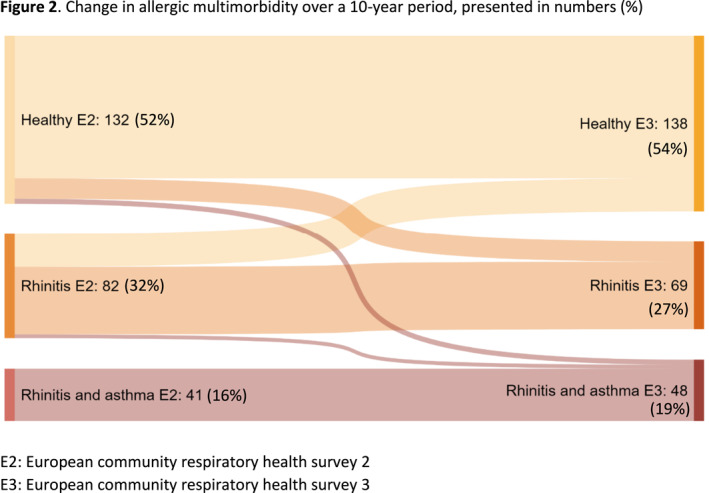
Change in allergic multimorbidity over 10 years, presented in numbers (%).

The study was based on participants from the European Community Respiratory Health Survey (ECRHS), a multicentre study with three visits: 1991–1992, 1999–2000 and 2011–2012. The study design of ECRHS I, II and III has been published before.^23^ Potential participants were sent a brief questionnaire in stage 1, and a random sample of those that responded was invited for a more detailed clinical examination (Stage 2). Additional subjects who reported symptoms of waking with shortness of breath, asthma attacks or using asthma medication in Stage 1 were also included. Individuals who participated in ECRHS I were invited to participate in ECRHS II and later in ECRHS III. The subjects answered a standardised questionnaire with the help of trained interviewers. The participants underwent lung function and blood tests to analyse inflammatory markers. In Uppsala, 824 individuals participated in ECRHS I, and 679 in ECRHS II and of these specific IgE measurements using microarray chip technology was done in 475. ECHRS III comprised 422 participants. This study included 255 individuals participating in both ECRHS II and ECRHS III, where specific IgE measurement was done in ECRHS II (Figure 1). The participants were grouped in terms of the presence or absence of rhinitis and asthma in ECRHS II and 10 years later in ECRHS III (Figure 2). In this grouping six individuals, that made up 4.6% of all participants with only asthma and no rhinitis, were excluded due to small sample size.

### 2.2 Questionnaires

The ECRHS II and ECRHS III main questionnaires (http://www.ecrhs.org)^24^ were used to obtain information about background factors, smoking history, parental allergy and asthma.

Rhinitis was defined as having had problems sneezing, or a runny or blocked nose when not having a cold in the last 12 months.^25^

Asthma was defined as ever being diagnosed with asthma and having had an asthma attack and one of the following symptoms during the past 12 months: nocturnal cough, attacks of breathlessness during rest, following activity or at night, chest wheezing, or whistling, taking asthma medication.^26^

Eczema was defined as having an itchy rash that came and went for at least 6 months and having had this problem within the past 12 months.^27^

### 2.3 Allergy testing

The presence of IgE antibodies was examined using microarray chip technology (ImmunoCAP ISAC; Thermo Fischer Scientific, Uppsala, Sweden).^28^, ^29^ The chip had 103 native or recombinant allergen components from 43 allergen sources. Specific IgE was reported as ISAC Standardised Units (ISU), which is a semi‐quantitative estimate of the actual specific IgE levels. Subjects were regarded as being non‐IgE sensitised if the signal could not be measured or was very low (<0.3 ISU). Allergen component analysis was performed in 475 individuals in ECRHS II. All 255 participants in this study had allergen component analysis available for ECRHS II.

### 2.4 Inflammatory markers

Total serum IgE for both ECRHS II and III was measured using the Pharmacia CAP system (Uppsala, Sweden). Total IgE was available for 470 individuals in ECRHS II and 351 in ECRHS III. Of the participants in our study 248 had results available in ECRHS II and 217 in ECHRS III.

F_E_NO measurements were made using an exhalation flow rate of 50 ml/s.^30^ The system used for NO measurements in ECRHS II was a computer‐based single‐breath NO system from Nitrograf (Hässelby, Sweden) that used a chemiluminescence analyser (Sievers NOA 280; Sievers, Boulder, Col, USA). In ECRHS III, F_E_NO was measured using an electrochemical analyser (NIOX MINO; Aerocrine AB).^31^ F_E_NO was measured in 288 people in ECRHS II and 361 in ECRHS III. In the current study, 156 participants had available results in ECRHS II and 223 in ECRHS III.

Blood samples were collected for ECP. Samples were kept at 24°C for 60 min before centrifugation. In ECRHS II, the concentration of serum ECP was assayed with Pharmacia ECP RIA (Pharmacia Diagnostics) and in ECRHS III, with ImmunoCAP ECP (Thermo Fischer Scientific). ECRHS III also included blood samples for serum EDN, which were measured using an ImmunoCAP research assay.^32^ ECP was analysed in 409 individuals in ECRHS II and ECP and EDN in 321 people in ECRHS III. In the current study 212 participants had ECP available in ECRHS II and 207 had ECP and EDN available in ECRHS III.

### 2.5 Lung function

Forced expiratory volume in one second (FEV_1_) was measured in ECRHS II using a dry rolling‐seal spirometer (Model 2130; SensorMedics, Anaheim, Cal, USA). Up to five technically acceptable manoeuvres were measured. American Thoracic Society recommendations were followed.^33^ The predicted values for FEV_1_ were calculated on the basis of European Coal and Steel Union reference values.^34^ Weight and height were measured, and body mass index (BMI) was calculated.

### 2.6 Statistical methods

Statistical analyses were performed using STATA (14.2; StataCorp, College Station, Tex). Non‐normally distributed variables, F_E_NO, total IgE and ECP, EDN were log‐transformed before analysis. The *χ* ^2^ test or Fishers' exact test, in case of low prevalence in groups (<5 individuals), and analysis of variance (ANOVA) with Bonferroni correction were used when comparing groups. Logistic regression and multinominal logistic regression were used in the multivariable analysis. A *p*‐value of <0.05 was considered statistically significant.

### 2.7 Ethics

The study was approved by the Regional Ethical Review Board in Uppsala, Sweden (Dnr for ECHRS II and III was 1999/313, 2010/068 respectively). Informed consent was obtained from all individual participants included in the study.

## 3 RESULTS

Over 10 years, comparing ECRHS II and ECRHS III, we found that of the 132 healthy individuals at baseline, 112 remained healthy, 16 developed rhinitis and 4 asthma and rhinitis (Figure 2). Out of 82 subjects with rhinitis, 26 went into remission, 53 remained unchanged and 3 developed asthma in addition to rhinitis (Figure 2). None of the 41 participants with asthma and rhinitis went into remission.

Participants with persistent rhinitis and asthma were younger compared with healthy individuals (Table 1). Subjects with persistent disease were likelier to have heredity for allergic disease and asthma. Individuals with persistent disease, but mainly concomitant asthma and rhinitis, had lower lung function at inclusion in ECRHS II. No difference was seen with regard to gender, BMI or smoking status.

**TABLE 1:** Demographics, background factors and lung function in ECRHS II. Grouped according to disease status in ECRHS II and III, mean ± SD and *n* (%).^a^

	Healthy (*n* = 112)	New onset rhinitis (*n* = 16)	Remission of rhinitis (*n* = 26)	Persistent rhinitis (*n* = 53)	Persistent rhinitis and asthma (*n* = 41)	*p*‐value
Age (years)	44 ± 7	43.8 ± 8	41.4 ± 7	43.4 ± 7	39.2 ± 7	0.006
Woman	52 (46)	4 (25)	14 (54)	23 (43)	22 (54)	0.332
FEV_1_ (%pred)	108.4 ± 12	104.6 ± 17	109.1 ± 11	108.7 ± 14	96.9 ± 16	<0.001
BMI (kg/m^2^)	25 ± 4	26 ± 5	23 ± 3	24.8 ± 5	25 ± 4	0.536
Eczema during the last 12 months	14 (13)	6 (38)	9 (35)	10 (19)	16 (40)	0.001
Never smokers	55 (49)	11 (69)	17 (65)	25 (47)	28 (68)	0.074
Ex‐smokers	47 (42)	3 (19)	6 (23)	24 (45)	7 (17)	
Current smokers	10 (9)	2 (13)	3 (12)	4 (8)	6 (15)	
Total IgE^b^	20.2 (16.2–25.2)	43.9 (19.2–100.5)	23.8 (12–43.5)	47.3 (32.7–68.3)	115 (76.2–173)	<0.001
ECP^b^	6.97 (6.24–7.79)	6.19 (4.13–9.29)	7.33 (5.47–9.83)	8.93 (7.29–10.9)	8.62 (7.31–10.2)	0.075
Heredity for allergy						0.036
No heredity	69 (53)	9 (7)	15 (12)	26 (20)	12 (9)	
Heredity from 1 parent	38 (38)	7 (7)	10 (10)	23 (23)	23 (23)	
Heredity from both parents	5 (31)	0	1 (6)	4 (25)	6 (38)	
Heredity for asthma						0.005
No heredity	102 (49)	15 (7)	19 (9)	44 (21)	28 (13)	
Heredity from 1 parent	10 (25)	1 (3)	7 (18)	9 (23)	13 (33)	
Heredity from both parents	No observations					

Abbreviations: ECP, eosinophil cationic protein; ECRHS, European Community Respiratory Health Survey; IgE, immunoglobulin E.

^a^ A total of seven individuals were not included in the table due to the small group size. (Healthy developing rhinitis/asthma *n* = 4, rhinitis developing rhinitis/asthma *n* = 3).

^b^ Presented as geometric mean and 95% confidence interval.

### 3.1 Sensitisation

**FIGURE 3:**
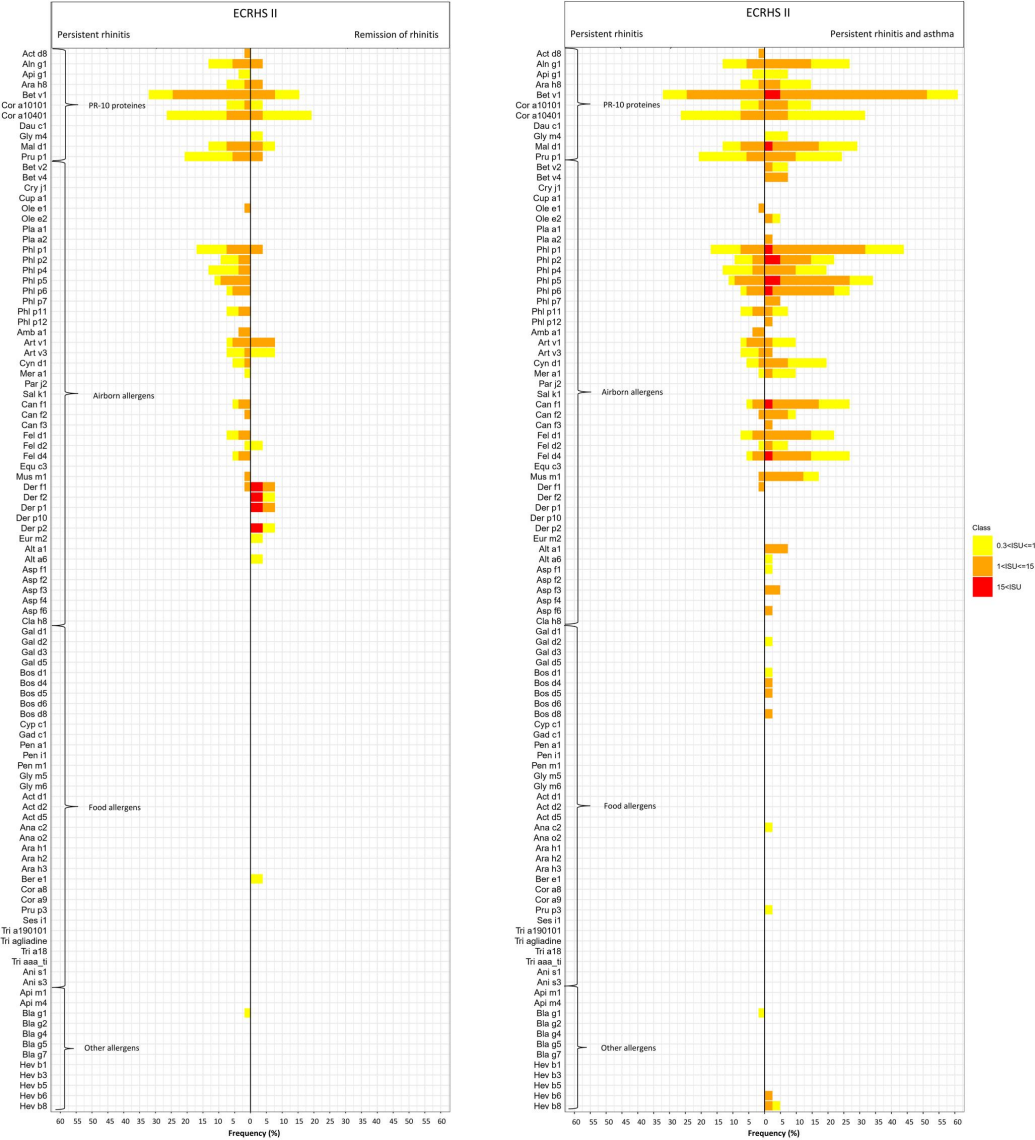
Sensitisation to allergen components according to microarray chip technology (ImmunoCAP ISAC) in ECRHS II regarding the change in rhinitis and asthma. Yellow < 0.3–1 ISU; orange 1–15 ISU; red > 15 ISU.

Participants with persistent rhinitis were more likely to be sensitised to any allergen, food of plant origin, grass and tree pollen at baseline (Tables 2 and 3) than individuals that were healthy both at baseline and at follow‐up. Subjects with persistent rhinitis were more likely to be sensitised to grass pollen than those that went into remission (Table 3, Figure 3). Individuals with persistent asthma were more likely to be sensitised to tree pollen and furry animals than those with only persistent rhinitis (Table 4, Figure 3).

**TABLE 2:** Sensitisation at ECRHS II to allergen groups in terms of change in allergic multimorbidity over a 10‐year period, presented in *n* (%).^a^

	Healthy (*n* = 112)	New onset of rhinitis (*n* = 16)	Remission of rhinitis (*n* = 26)	Persistent rhinitis (*n* = 53)	Persistent rhinitis and asthma (*n* = 41)	*p*‐value
Foods of plant origin	16 (14.3)	2 (12.5)	6 (23.1)	17 (32.1)	20 (48.8)	<0.001
Foods of animal origin	2 (1.8)	1 (6.3)	0	0	1 (2.4)	0.358
Grass pollen	3 (2.7)	4 (25)	1 (3.9)	13 (24.5)	21 (51.2)	<0.001
Tree pollen	6 (5.4)	7 (43.8)	4 (15.4)	17 (32.1)	26 (63.4)	<0.001
Weed pollen	2 (1.8)	0	2 (7.7)	5 (9.4)	4 (9.8)	0.066
Furry animals	1 (0.9)	1 (6.3)	1 (3.9)	6 (11.3)	19 (46.3)	<0.001
Mites	0	1 (6.3)	3 (11.5)	1 (1.9)	0	0.003
Mould	0	0	1 (3.9)	0	5 (12.2)	0.001
Cockroach	2 (1.8)	0	0	1 (1.9)	0	1
Latex	0	1 (6.3)	3 (11.5)	1 (1.9)	0	0.003
Other	0	2 (12.5)	0	1 (1.9)	5 (12.2)	<0.001
Sensitised to any allergen	24 (21.4)	10 (62.5)	11 (42.3)	31 (58.5)	31 (75.6)	<0.001
Number of positive components, median (IQ range)	0	1 (0–4)	0 (0–1)	1 (0–4)	5 (0.5–10)	<0.001

Abbreviation: ECRHS, European Community Respiratory Health Survey.

^a^A total of seven individuals were not included in the table due to the small group size. (Healthy developing rhinitis/asthma *n* = 4, rhinitis developing rhinitis/asthma *n* = 3).

**TABLE 3:** Multivariable analysis comparing healthy participants with those with persistent rhinitis and those with remission of rhinitis, presented as risk ratio, 95% confidence interval.

	Remission of rhinitis, *n* = 26	Persistent rhinitis, *n* = 53	*p*‐value^a^
Age^b^	0.97 (0.91–1.03)	1 (0.96–1.06)	0.25
FEV_1_%^b^ ^,^ ^c^	0.99 (0.68–1.44)	1.05 (0.78–1.41)	0.78
Eczema^b^	3.29 (1.17–9.22)	1.53 (0.59–4.01)	0.18
No heredity for allergy^b^	1	1	
Heredity from one parent^b^	0.93 (0.36–2.39)	1.35 (0.64–2.84)	0.47
Heredity from both parents^b^	0.88 (0.09–8.72)	1.94 (0.43–8.75)	0.51
Sensitisation to foods of plant origin, E2^d^	1.79 (0.59–5.42)	2.98 (1.32–6.74)	0.37
Sensitisation to grass pollen, E2^d^	1.22 (0.12–12.7)	11.9 (3.16–45.1)	0.04
Sensitisation to tree pollen, E2^d^	2.62 (6.42–10.7)	7.96 (2.85–22.3)	0.09
Sensitisation to weed pollen, E2^d^	3.15 (0.38–26.3)	5.36 (0.98–29.2)	0.57
Sensitisation to furry animals, E2^d^	3.11 (0.17–55.1)	13.8 (1.58–120.3)	0.20
Sensitised to any allergen, E2^d^	2.61 (1.02–6.64)	5.22 (2.53–10.8)	0.17
Total IgE E2^d^ (per one log unit increase)	1.41 (0.62–3.2)	4.07 (1.99–8.32)	0.02
Total IgE E3^d^ (per one log unit increase)	1.3 (0.61–2.77)	3.45 (1.74–6.84)	0.03
F_E_NO E2^d^ (per one log unit increase)	2.51 (0.32–19.9)	2.13 (0.41–11.1)	0.89
F_E_NO E3^d^ (per one log unit increase)	0.69 (0.09–5.42)	1.88 (0.34–10.4)	0.40
ECP E2^d^ (per one log unit increase)	1.90 (0.29–12.6)	6.80 (1.35–34.2)	0.25
ECP E3^d^ (per one log unit increase)	1.09 (0.23–5.16)	0.99 (0.28–3.56)	0.92
EDN E3^d^ (per one log unit increase)	1.33 (0.24–7.36)	1.36 (0.34–5.50)	0.98

*Note*: Inflammatory markers were log transformed.

Abbreviations: E2, European community respiratory health survey II; E3, European community respiratory health survey III; ECP, eosinophil cationic protein; EDN, eosinophil derived neurotoxin; F_E_NO, exhaled nitric oxide; IgE, immunoglobulin E.

^a^ *p*‐value when comparing those with remission with those with persistent rhinitis.

^b^ Adjusted for age, FEV1, current eczema, heredity for allergy and being sensitised to any allergen.

^c^ Change calculated in 10% decrease intervals in FEV1.

^d^ Adjusted for age, FEV1, current eczema, heredity for allergy.

**TABLE 4:** Multivariable analysis comparing those with persistent rhinitis to those with persistent asthma and rhinitis, presented as odds ratio, 95% confidence interval.

	OR (95% CI)
Age^a^	0.94 (0.88–1.01)
FEV_1_%^a^ ^,^ ^b^	0.56 (0.40–0.80)
Eczema^a^	2.24 (0.76–6.55)
No heredity for allergy^a^	1
Heredity from one parent^a^	1.35 (0.48–3.79)
Heredity from both parents^a^	1.72 (0.28–10.7)
Sensitisation to foods of plant origin, E2^c^	1.40 (0.53–3.70)
Sensitisation to grass pollen, E2^c^	2.60 (0.95–7.25)
Sensitisation to tree pollen, E2^c^	3.50 (1.29–9.49)
Sensitisation to weed pollen, E2^c^	0.55 (0.11–2.76)
Sensitisation to furry animals, E2^c^	6.73 (2.00–22.6)
Sensitisation to mould, E2^c^	1
Sensitised to any allergen, E2^c^	1.17 (0.39–3.49)
Total IgE E2^c^ (per one log unit increase)	2.46 (0.99–6.12)
Total IgE E3^c^ (per one log unit increase)	1.55 (0.61–3.98)
F_E_NO E2^c^ (per one log unit increase)	16.2 (1.14–230)
F_E_NO E3^c^ (per one log unit increase)	3.76 (0.48–29.4)
ECP E2^c^ (per one log unit increase)	0.72 (0.08–6.08)
ECP E3^c^ (per one log unit increase)	4.44 (0.59–33.6)
EDN E3^c^ (per one log unit increase)	2.80 (0.30–26.4)

*Note*: Inflammatory markers were log transformed.

Abbreviations: CI, confidence interval; E2, European community respiratory health survey II; E3, European community respiratory health survey III; ECP, eosinophil cationic protein; EDN, eosinophil derived neurotoxin; F_E_NO, exhaled nitric oxide; IgE, immunoglobulin E; OR, odds ratio.

^a^ Adjusted for age, FEV1, current eczema, heredity for allergy and being sensitised to any allergen.

^b^ Change calculated in 10% decrease intervals in FEV1.

^c^ Adjusted for age, FEV1, current eczema, heredity for allergy.

### 3.2 Inflammatory markers

Participants with persistent rhinitis had higher levels of total IgE in ECRHS II and 10 years later in ECRHS III, compared with both healthy individuals and those with remission of rhinitis (Table 3, online supplement Table 1E, online supplement Figure 1E). Levels of ECP were elevated at baseline when comparing healthy individuals with those with persistent rhinitis (Table 3). Subjects with persistent rhinitis and asthma had higher levels of F_E_NO at baseline than those with only persistent rhinitis (Table 4). Subjects with persistent rhinitis and asthma had higher levels of total IgE at baseline and after 10 years, as well as F_E_NO and ECP at baseline, compared with those that remained healthy (Online supplement Table 2E). No difference was seen in F_E_NO, ECP and EDN in ECRHS III between those with persistent rhinitis and those that went into remission or remained healthy (Table 3, online supplement Figure 1E). The above‐mentioned findings persisted after adjusting for age, FEV_1_, eczema and allergic heredity (Tables 3 and 4 and Online supplement Table 2E).

## 4 DISCUSSION

Our main findings were that those with persistent rhinitis over 10 years were more likely to be sensitised to allergens and have higher specific IgE levels to allergen components than those that remained healthy or went into remission. Subjects with persistent rhinitis had higher levels of total IgE both at baseline and after 10 years compared with those that remained healthy or went into remission. Those with persistent rhinitis had higher levels of ECP at baseline compared with individuals that remained healthy. Participants with persistent asthma had higher levels of F_E_NO at baseline compared with those with persistent rhinitis. In the study, those with asthma and rhinitis retained their disease status over time. In contrast, some change was seen among individuals that were initially healthy or only had rhinitis at baseline.

### 4.1 Allergic sensitisation

In the present study, persistent rhinitis was associated with allergic sensitisation and a higher number and level of sensitisation to allergen components. It has previously been observed that remission of allergic rhinitis or asthma is more frequent in those with lower levels of allergen‐specific IgE antibodies.^35^, ^36^, ^37^ We found that both seasonal and perennial allergen sensitisation was associated with persistent allergic disease. Some studies note sensitisation to seasonal allergens as being more important in continued allergic rhinitis,^35^ whilst others also found an association with perennial allergens.^36^

Treatment with allergen‐specific immunotherapy in childhood has previously been shown to reduce the development of allergic disease later in life.^38^ Allergen immunotherapy has been shown to reduce symptoms of allergic rhinitis and asthma and use of medication, sometimes down to no medication at all.^39^

### 4.2 Type 2 inflammation

We found that total IgE levels both at baseline and after 10 years were higher among those with persistent rhinitis than those that remained healthy or went into remission. It has previously been shown that those with allergic rhinitis have higher total IgE levels compared with healthy individuals.^40^, ^41^ Elevated levels of total IgE in cord blood at birth have been described as a risk factor for developing asthma and rhinitis later in life.^42^, ^43^ Elevated total IgE in early childhood has been associated with food and mite sensitisation and a higher risk for developing asthma and rhinitis later in childhood.^44^ Little data exists on the predictive value of total IgE on retaining allergic rhinitis in adulthood.

We found that elevated levels of ECP at baseline correlated with persistent rhinitis compared with those that remained healthy. A small study showed high levels of both ECP and eosinophil peroxidase as predictive for the future development of asthma among those with allergic rhinitis.^45^ To our knowledge, no information on ECP as a predictive biomarker for persistent rhinitis is available.

F_E_NO at baseline was higher among those with persistent asthma than those with persistent rhinitis. It has previously been shown that individuals with asthma have higher levels of F_E_NO than those with only rhinitis.^40^ We also found that participants with persistent allergic disease had a higher baseline F_E_NO than those that remained healthy. Elevated levels of F_E_NO have been associated with both persistent and new onset rhinitis as well as the development of asthma over time.^16^, ^46^

There was no group difference in EDN levels in ECRHS III. It has previously been described that elevated levels of EDN in children with wheezing could predict the later development of asthma.^21^ Unfortunately, no serum EDN was measured in ECRHS II.

### 4.3 Age and heredity

We found that those with persistent asthma and rhinitis were more likely to be younger and have heredity for both allergy and asthma. Previous studies have shown that remission of adult‐onset asthma is rare,^7^ whereas asthma starting before age 10 is more likely to go into remission.^8^ A positive family history for allergic disease is a known risk factor for having allergic rhinitis and asthma^15^ and is described to be negatively associated with remission.^8^, ^47^, ^48^

Those with persistent asthma and rhinitis had lower lung function. It has previously been shown that asthmatics with nasal disease have lower lung function.^49^

### 4.4 Strengths and weaknesses

The study is population‐based and longitudinal, enabling comparison of the same individuals over time. Trained investigators conducted interviews. An extensive specific IgE analysis was performed using multiplex allergen component analysis which minimises the risk of missing allergen sensitisations.

Incorporating asthma diagnosis into the definition can be considered both a strength and weakness, as we likely include true asthmatics but might have missed some individuals who have asthma but lack a formal diagnosis.

A limitation is that the group categorisation was based on self‐reported data, and the relatively small sample size could affect the statistical results. Type‐2 inflammation markers were not available for all participants, and EDN levels were not available in ECRHS II for comparison at baseline. Different measuring devices were used for F_E_NO in ECRHS II and ECRHS III, which might impact the results.

## 5 CONCLUSION

IgE sensitisation and total IgE levels are associated with persistence of rhinitis and having both asthma and rhinitis. Participants with persistent allergic disease had higher levels of allergen sensitisation and type 2 inflammation markers at baseline compared with those that remained healthy. Identifying individuals at risk for continued rhinitis and asthma allows clinicians to better plan follow‐up appointments and improve the selection of patients who might benefit from allergen immunotherapy or treatments targeting type‐2 inflammation.

## AUTHOR CONTRIBUTIONS

Viiu Blöndal drafted the manuscript, performed the data analysis and interpretation with the help of Christer Janson. Christer Janson, Fredrik Sundbom, Andrei Malinovschi, Kjell Alving, Marieann Högman, Magnus P. Borres, Robert Movérare and Xingwu Zhou reviewed the manuscript. Xingwu Zhou made Figure 3. All authors read and approved the final manuscript.

## CONFLICT OF INTEREST STATEMENT

Magnus P. Borres and Robert Movérare are affiliated with Thermofisher Scientific (Sweden) that also provided financial support for this study. Kjell Alving received research material from Hemocue AB, Thermo Fisker Scientific, NIOX Group Plc. No other author has reported any conflict of interest.

## Supporting information

Supporting Information S1

Supporting Information S1

## Data Availability

The dataset is held and managed by the Department of Medical Sciences, Uppsala University, Uppsala, Sweden. Data cannot be made freely available as they are subject to secrecy in accordance with the Swedish Public Access to Information and Secrecy Act, but can be made available to researchers upon request (subject to a review of secrecy). Requests for data can be sent to the Unit for Respiratory, Allergy and Sleep Research at the University Hospital in Uppsala lungforskning@akademiska.se.
