# Supplementary material for: Allergic sensitisation and type‐2 inflammation is associated with new‐onset and persistent allergic disease
Source: Clin Transl Allergy. 2023 Apr 6;13(4):e12240. doi: 10.1002/clt2.12240 (PMC10080081; doi:10.1002/clt2.12240)
Supplement: Supplementary file 1 — Supporting Information S1 [file CLT2-13-e12240-s001.pdf]

Online supplement figure 1e

Allergic disease status in terms of inflammatory markers in ECRHS II and III

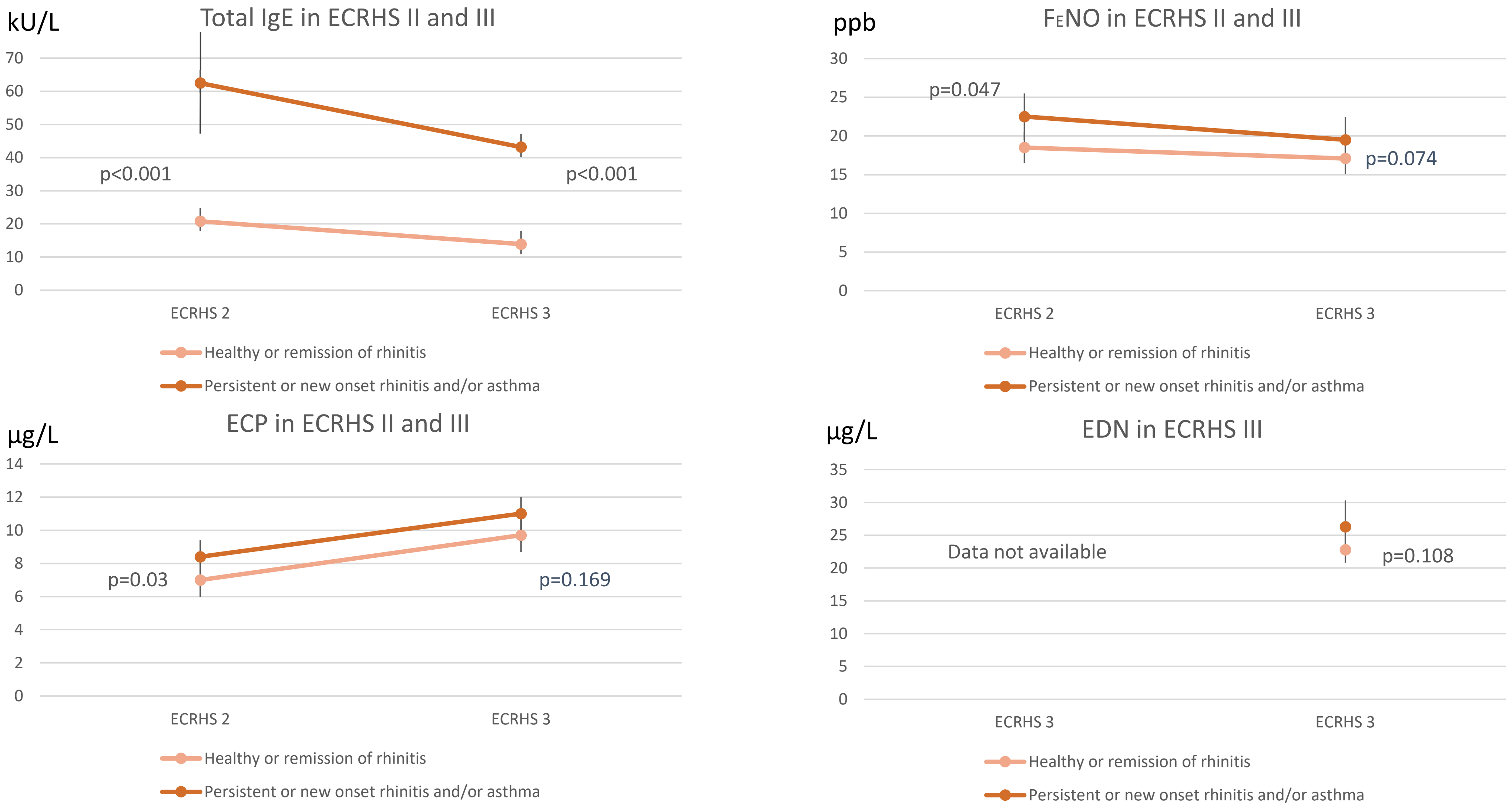

ECRHS: European community respiratory health survey

IgE: Immunoglobulin E

F_ENO: Exhaled nitric oxide

ECP: Eosinophil cationic protein

EDN: Eosinophil derived neurotoxin
