# Supplementary material for: Allergic sensitisation and type‐2 inflammation is associated with new‐onset and persistent allergic disease
Source: Clin Transl Allergy. 2023 Apr 6;13(4):e12240. doi: 10.1002/clt2.12240 (PMC10080081; doi:10.1002/clt2.12240)
Supplement: Supplementary file 2 — Supporting Information S1 [file CLT2-13-e12240-s002.docx]

| **Online supple Table 1e.** Levels of type 2 inflammatory markers in ECRHS II and ECRHS III in terms of change in allergic multimorbidity over time. Presented as geometric mean (confidence interval) | | | |
| --- | --- | --- | --- |
|  | Healthy (n=112) | Remission of rhinitis (n=26) | Persistent rhinitis (n=53) |
| Total IgE in E2 (kU/L) | 20.2 (16.2– 25.2) | 23.8 (13–43.5) | 47.3 (32.7–68.3) |
| Total IgE in E3 (kU/L) | 13.6 (10.3–18.0) | 15.3 (8.3–28.1) | 33.5 (22.9–49.1) |
| F_E_NO in E2 (ppb) | 18.1 (16.1–20.3) | 20.9 (13.8–31.5) | 19.7 (15.8–24.6) |
| F_E_NO in E3 (ppb) | 17.3 (15.8–19.1) | 16.0 (12.6–20.3) | 18.2 (15.8–20.9) |
| ECP in E2 (µg/L ) | 7.0 (6.2–7.8) | 7.3 (5.5–9.8) | 8.9 (7.3–10.9) |
| ECP in E3 (µg/L ) | 9.6 (8.4–10.9) | 10.0 (7.4–13.4) | 9.7 (7.4–12.6) |
| EDN in E3 (µg/L ) | 22.6 (20.0–25.7) | 23.7 (18.1–31.0) | 23.8 (19.2–29.5) |
| E2: European community respiratory health survey II  E3: European community respiratory health survey III  IgE: Immunoglobulin E  F_E_NO: Exhaled nitric oxide  ECP: Eosinophil cationic protein  EDN: Eosinophil derived neurotoxin | | | |

| **Online supple Table 2e.** Multivariable analysis comparing participants with persistent rhinitis and or asthma to participants that remain healthy | |
| --- | --- |
|  | aOR (95% CI) |
| Age^A^, | 1.00 (0.95-1.04) |
| FEV_1_%^A^ | 0.84 (0.66-1.06) |
| Eczema ^A^ | 2.20 (0.95-5.07) |
| No heredity for allergy ^A^ | 1 |
| Heredity from 1 parent ^A^ | 1.52 (0.78-2.98) |
| Heredity from both parents ^A^ | 2.30 (0.62-8.52) |
| Sensitisaiton to foods of plant origin, E2^B^ | 3.40 (1.66-7.02) |
| Sensitisation to grass pollen, E2^B^ | 16.7 (4.78-58.3) |
| Sensitisation to tree pollen, E2^B^ | 12.9 (5.02-33.4) |
| Sensitisation to weed pollen, E2^B^ | 3.67 (0.73-18.4) |
| Sensitisation to furry animals, E2^B^ | 32.6 (4.20-253) |
| Sensitised to any allergen, E2^B^ | 6.16 (3.05-11.9) |
| Total IgE E2^B^ (per one log unit increase) | 6.16 (3.05-12.5) |
| Total IgE E3^B^ (per one log unit increase) | 4.44 (2.29-8.61) |
| F_E_NO E2^B^ (per one log unit increase) | 5.21 (1.20-22.7) |
| F_E_NO E3^B^ (per one log unit increase) | 2.93 (0.77-11.2) |
| ECP E2^B^ (per one log unit increase) | 6.32 (1.52-26.4) |
| ECP E3^B^ (per one log unit increase) | 1.71 (0.58-5.06) |
| EDN E3^B^ (per one log unit increase) | 2.02 (0.62-6.52) |
| ^A^ Adjusted for age, FEV1, current eczema, heredity for allergy and being sensitised to any allergen  ^B^ Adjusted for age, FEV1, current eczema, heredity for allergy  E2: European community respiratory health survey II  Inflammatory markers were log transformed  E3: European community respiratory health survey III  IgE: Immunoglobulin E  F_E_NO: Exhaled nitric oxide  ECP: Eosinophil cationic protein  EDN: Eosinophil derived neurotoxin | |
